# Association Analysis of ENPP1 Tissue Expression, Polymorphism, and Growth Traits in Xiangsu Pigs

**DOI:** 10.3390/genes16040395

**Published:** 2025-03-29

**Authors:** Jiaqi Chen, Jiajin Huang, Houqiang Xu

**Affiliations:** 1 Key Laboratory of Animal Genetics, Breeding and Reproduction in the Plateau Mountainous Region, Ministry of Education, Guizhou University, Guiyang 550025, China; jqchen1@gzu.edu.cn (J.C.); jihqwe023642@163.com (J.H.); 2 College of Animal Science, Guizhou University, Guiyang 550025, China

**Keywords:** ENPP1, growth properties, SNPs, Xiangsu hybrid pig

## Abstract

**Background:** Pigs are vital agricultural animals, with growth traits serving as key indicators of their quality. **Methods:** In this study, we examined the mRNA expression of ENPP1 as a candidate gene in heart, liver, spleen, lungs, and kidneys at 3 days and 6 months of age by real-time polymerase chain reaction method and single-nucleotide polymorphism (SNP) loci in 165 Xiangsu pigs by Sanger sequencing. **Results:** The expression of ENPP1 in different tissues of Xiangsu pigs at different stages was significantly different, and it had high conservation in different species.. Sequence alignment with reference data identified five SNP sites: g.64275T→C and g.64429G→A in intron 19, g.64850T→C and g.64911G→A in intron 20, and g.64527T→C in exon 20. Association analysis revealed that g.64275T→C, g.64429G→A, and g.64527T→C significantly influence the growth performance of Xiangsu pigs (*p* < 0.05). **Conclusions:** These findings suggest that ENPP1 polymorphisms are closely associated with growth traits in Xiangsu pigs and may provide valuable insights for molecular breeding of this breed.

## 1. Introduction

Pigs are vital livestock, providing a rich source of meat and serving as a key food resource for humans. Indigenous Chinese pig breeds exhibit unique genetic traits, making them an important part of global germplasm resources [1]. The Congjiang Xiang pig, a small native breed from Guizhou, China, is renowned for its strong resilience and tender, flavorful meat; however, it has relatively slow growth and development. To overcome this limitation, the Xiangsu hybrid pig was developed through successive backcrossing. The Congjiang Xiang pig was used as the maternal line, while the Suzhou Taihu pig served as the paternal line. Subsequent generations were then backcrossed to the Congjiang Xiang pig to maintain the maternal genetic contribution [2]. This hybrid combines the tender, flavorful meat and strong adaptability of the Congjiang Xiang pig with the rapid growth rate and high litter size of the Suzhou Taihu pig [3]. In this study, by using ENPP1 as a candidate gene, detecting mRNA expression in the heart, liver, spleen, lungs, and kidneys at 3 days and 6 months of age by real-time polymerase chain reaction and analyzing the relationship between different genotypes of ENPP1 in Xiangsu crossbred pigs and growth traits at 6 months of age by Sanger sequencing, we identified five SNPs in the ENPP1 gene of Xiangsu pigs and screened them to find out the superior genes or genotypes of superior growth traits and predicted the functions of the ENPP1 gene by using bioinformatics analysis technology. Finally, we carried out a correlation analysis between the growth traits and mutation sites in Xiangsu crossbred pigs, which will provide a theoretical basis for the subsequent investigation of molecular genetic breeding of Xiangsu crossbred pigs.

Ecto-nucleotide pyrophosphatase/phosphodiesterase 1 (ENPP1) is a type II transmembrane protein that belongs to the ecto-nucleotide pyrophosphatase/phosphodiesterase (ENPP) family [4]. To date, seven family members (ENPP1–7) have been identified, all of which share a conserved catalytic core, the phosphodiesterase (PDE) domain [5]. In ENPP1–3, the PDE domain is flanked by two N-terminal auxin-binding protein-like domains and one inactive nuclease domain at the C-terminus. In contrast, ENPP4–7 contain only the PDE domain. Structural variations in the substrate-binding sites confer distinct properties to each protein. Consequently, ENPPs 1, 3, 4, and 5 hydrolyze nucleotides, while ENPPs 2, 6, and 7 have evolved into phosphodiesterases through their catalytic domains [6,7]. As the founding member of the ENPP family, ENPP1 regulates skeletal and soft tissue mineralization by generating inorganic pyrophosphate (PPi). Studies have shown that ENPP1 catalyzes the hydrolysis of ATP or GTP, producing PPi, which subsequently influences bone and cartilage mineralization. This positions ENPP1 as a central regulator of skeletal and cartilage development in mammals [8]. ENPP1 knockout mouse models display severe ectopic calcification and bone defects in multiple tissues, accompanied by the progressive loss of osteocytes [9,10]. Furthermore, ENPP1 functions as an inhibitor in the insulin signaling pathway, modulating biological processes related to obesity and type 2 diabetes (T2D) by influencing insulin resistance (IR) in tissues and cells. Overexpression of ENPP1 in adipocytes has been shown to promote the development of conditions such as fatty liver and hyperlipidemia [11,12,13]. In addition, numerous studies have demonstrated the widespread expression of ENPP1 in tissues, including bone, liver, skeletal muscle, ovaries, and adipose tissue. ENPP1 is essential for purinergic signaling and plays a key role in regulating neuroimmune responses, musculoskeletal functions, hormonal balance, and blood circulation [14,15,16]. In related studies reporting on whole genome resequencing and transcriptome analyses in the Min pig, the results showed that the ENPP1 gene is a candidate gene related to body size variation in Min pigs, but no further studies on ENPP1 gene were seen [17].

Alterations in factors such as selection rather than random mating, migration, mutation and natural selection can result in changes in the genetic balance of a population, and therefore mutations are important for the evolution of populations. Studies have demonstrated that gene polymorphisms and SNP sites regulate the growth and development of animals [18,19]. Investigating the SNP sites and polymorphisms within the ENPP1 gene will provide essential data for further studies on the role of ENPP1 in the growth traits of Xiangsu hybrid pigs. This study is the first to identify five SNPs in the ENPP1 gene of Xiangsu pigs and uses bioinformatics tools to predict the functional implications of these mutations. Finally, a correlation analysis was conducted between the growth traits of Xiangsu hybrid pigs and the identified mutations, laying a theoretical foundation for future molecular genetic breeding of this hybrid pig population.

## 2. Materials and Methods

### 2.1. Experimental Animals

The 165 Xiangsu pigs used in this study were provided by the Xiangsu Pig Breeding Farm of Guizhou University and were uniformly fed until 6 months of age, when blood samples were collected and growth traits were measured. The measured traits included withers height, body weight, body length, chest width, chest depth, chest girth, abdominal girth, rib girth, and hind limb girth. Of these, weight was measured on an electronic table scale and the rest of the indicators were measured with a soft tape measure. Blood samples were also collected. Subsequently, three piglets were randomly selected for slaughter from the 165, and tissue samples from the heart, liver, spleen, lungs, and kidneys were collected.

For comparison, a separate group of three healthy 3-day-old piglets from the outside experimental group was selected for slaughter and tissue sample collection, following the same procedures. The experimental animals were provided by the Breeding Pig Farm of Guizhou University, and all procedures were performed in strict accordance with the guidelines of the Guizhou University Animal Welfare Committee (EAE-GZU-2023-E079).

### 2.2. Whole-Blood DNA, Tissue RNA Extraction, and cDNA Synthesis

Genomic DNA was extracted from the anterior vena cava blood samples of Xiangsu pigs using a DNA extraction kit (Tiangen, Beijing, China). Total RNA was isolated from the heart, liver, spleen, lungs, and kidneys of Xiangsu pigs at various developmental stages using TRIzol reagent. The RNA concentration was adjusted to 1000 ng/μL, and cDNA was synthesized via reverse transcription under the following conditions: 37 °C for 2 min, 50 °C for 15 min, and 85 °C for 2 min, in a single cycle. The resulting cDNA was stored at −20 °C.

### 2.3. Primer Design

Based on the DNA (accession number: NC_010443.5) and RNA (accession number: XM_021087933.1) sequences of the pig ENPP1 gene published by NCBI, PCR and fluorescent primers were designed using Primer Premier 5.0 software. GAPDH was used as the internal reference gene for quantitative fluorescence, and primers were synthesized by Qingke Biotechnology (Chongqing, China). Primer information is provided in Table 1.

### 2.4. PCR Sequence Amplification and Real-Time Fluorescence Quantitative Analysis

Genomic DNA was amplified using PCR in a 20 µL reaction mixture, which consisted of 10 µL PCR Mix (Genstar, Beijing, China), 1 µL each of forward and reverse primers, 1 µL genomic DNA, and 7 µL double-distilled water (ddH_2_O). The amplification program began with an initial denaturation at 94 °C for 3 min, followed by 35 cycles of denaturation at 94 °C for 30 s, annealing at 63 °C for 30 s, and extension at 72 °C for 5 min, with a final hold at 4 °C. The PCR products were subjected to 1% agarose gel electrophoresis for analysis, and subsequently purified and sequenced by Qingke Biotechnology (Chongqing, China). For qRT-PCR, the reaction mixture was prepared in a total volume of 20 µL, containing 1 µL cDNA, 0.5 µL each of forward and reverse primers, 10 µL 2×RealStar Fast SYBR qPCR Mix, and 8 µL ddH_2_O. The amplification conditions were as follows: an initial denaturation at 95 °C for 2 min, followed by 40 cycles of denaturation at 95 °C for 15 s, annealing at 60 °C for 30 s, and extension at 72 °C for 30 s.

### 2.5. Bioinformatics Analysis

Amino acid sequences of the ENPP1 gene from 10 species were retrieved from the NCBI database. The species included are *Sus scrofa* (XP_020943592.1), *Gallus gallus* (XP_004940275.3), *Bos taurus* (NP_001193141.1), *Anas platyrhynchos* (XP_038032814.1), *Phacochoerus africanus* (XP_047614516.1), *Homo sapiens* (NP_006199.2), *Mus musculus* (NP_001295256.1), *Capra hircus* (XP_017909005.1), *Oryctolagus cuniculus* (NP_001076211.2), and *Danio rerio* (NP_001025339.1). A phylogenetic tree was constructed using the MEGA software (www.megasoftware.net, accessed on 24 March 2025), employing the neighbor-joining method. The MEME suite (http://meme.nbcr.net/, accessed on 24 March 2025) was used to analyze the structural features and functional aspects of the ENPP1 protein across these species.

### 2.6. Statistical Analysis

The sequencing results were analyzed using SeqMan software (Pro), and peak charts were examined to identify SNP sites for subsequent statistical analysis. Hardy–Weinberg equilibrium (HWE) was evaluated using the Chi-square (χ^2^) test. Genetic polymorphism parameters, including observed heterozygosity (Ho), expected heterozygosity (He), polymorphic information content (PIC), and effective allele number (Ne), were calculated. One-way analysis of variance (ANOVA) was performed using SPSS 25.0 to assess differences in growth performance among genotype groups. A general linear model (GLM) was applied in SPSS for association analysis, with genotype effect as a fixed factor and phenotype as the dependent variable. Multiple comparisons were conducted using Duncan’s method. The statistical model used was as follows: Y_ij_ = μ + G_i_ + e_ij_, where Y_ij_ represents the observed value of the growth trait, μ is the overall mean, G_i_ is the genotype effect, and e_ij_ is the random error. Multiple comparisons among different genotypes were adjusted using the Bonferroni method. The expression levels of ENPP1 in various tissues of Xiangsu pigs were quantified using the 2^−ΔΔCt^ method. Data are presented as means ± standard deviation, with all values rounded to two decimal places.

## 3. Results

### 3.1. Expression Levels of ENPP1 in Different Tissues of Xiangsu Pigs

**Figure 1:**
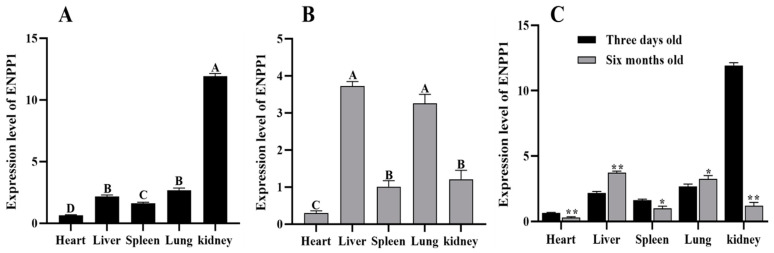
Expression level of ENPP1 in Xiangsu pigs. (**A**) Expression level of ENPP1 in tissues of 3-day-old Xiangsu pigs; (**B**) expression level of ENPP1 in tissues of 6-month-old Xiangsu pigs. * Indicating significant differences (*p*-value < 0.05); (**C**): Tissue expression of ENPP1 in different growth stages of Xiangsu pigs was different. ** different capital letters indicate extremely significant differences (*p*-value < 0.01).

Expression profiles of the ENPP1 gene in various tissues of 3-day-old and 6-month-old crispy pigs are shown in Figure 1. At 3 days, ENPP1 expression was highest in the kidneys and lowest in the heart. By 6 months of age, the highest expression was observed in the liver, while the heart exhibited the lowest expression (*p*-value < 0.01). Notable differences in ENPP1 expression were found across tissues at different ages. Specifically, 6-month-old Xiangsu pigs had higher expression levels in the liver and lungs compared to 3-day-old pigs, whereas expression was lower in the heart, spleen, and kidneys.

### 3.2. Analysis of ENPP1 Gene Polymorphism

**Figure 2:**
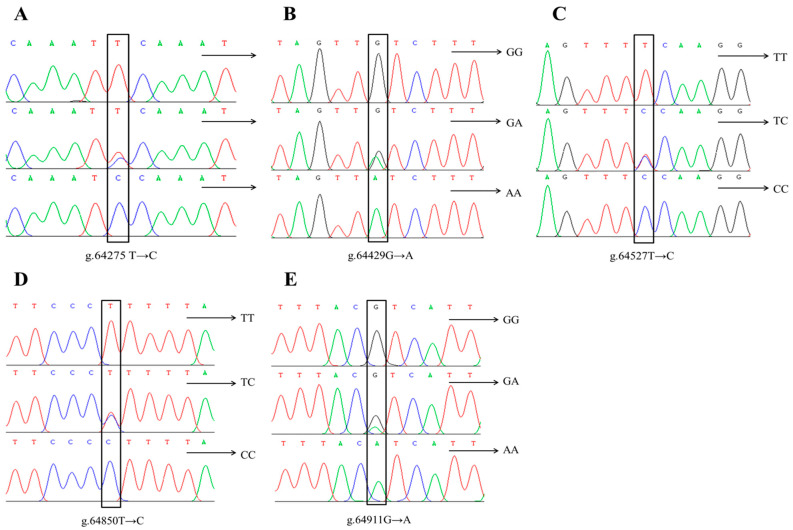
SNP loci of ENPP1 gene in Xiangsu pig. (**A**) g.64275 T→C, (**B**) g.64429G→A, (**C**) g.64527T→C, (**D**) g.64850T→C, (**E**) g.64911G→A.

The sequencing results were aligned with the pig ENPP1 reference sequence (accession number: NC_010443.5) using SeqMan software (Figure 2). Five SNPs were identified in the ENPP1 gene: g.64275T→C, g.64429G→A, g.64527T→C, g.64850T→C, and g.64911G→A. The g.64275T→C and g.64429G→A variants are located in intron 19, while g.64850T→C and g.64911G→A are found in intron 20, with g.64850T→C also present in exon 20. All five SNPs display two alleles and three genotypes, and all are synonymous mutations, meaning they do not alter the amino acid sequence.

### 3.3. Biodiversity Analysis

**Figure 3:**
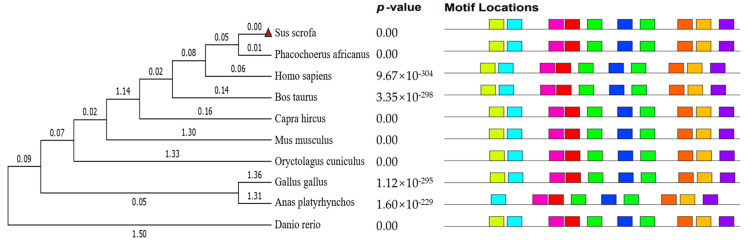
Evolutionary tree of the ENPP1 system for 10 species (**Left**) and motif structure analysis (**Right**).

**Figure 4:**
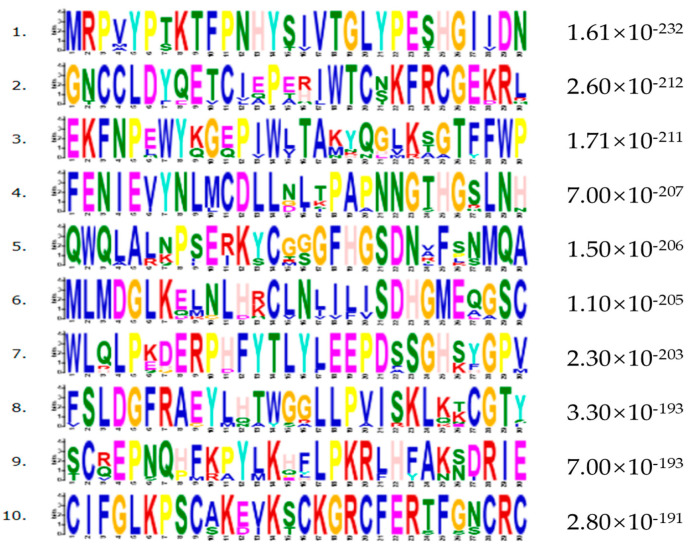
The motif sequence of ENPP1 in 10 species.

A phylogenetic tree of ENPP1 sequences from 10 species was constructed using MEGA6 software (6.0), based on a neighbor-joining analysis. The species included mammals (mouse, pig, cattle, goat, warthog, and rabbit), as well as human, chicken, duck, and zebrafish. Conserved motifs within the supersecondary structure were predicted using the MEME suite (http://meme-suite.org/tools/meme, accessed on 24 March 2025). The phylogenetic analysis showed that pigs are most closely related to warthogs, followed by humans and cattle, with zebrafish being the most distantly related (Figure 3). Ten significant amino acid sequences were identified across the ten species (Motif Count: 6–30), suggesting functional similarities at the supersecondary structure level (Figure 4).

### 3.4. Analysis of Growth Data of Xiangsu Hybrid Pig Population

**Figure 5:**
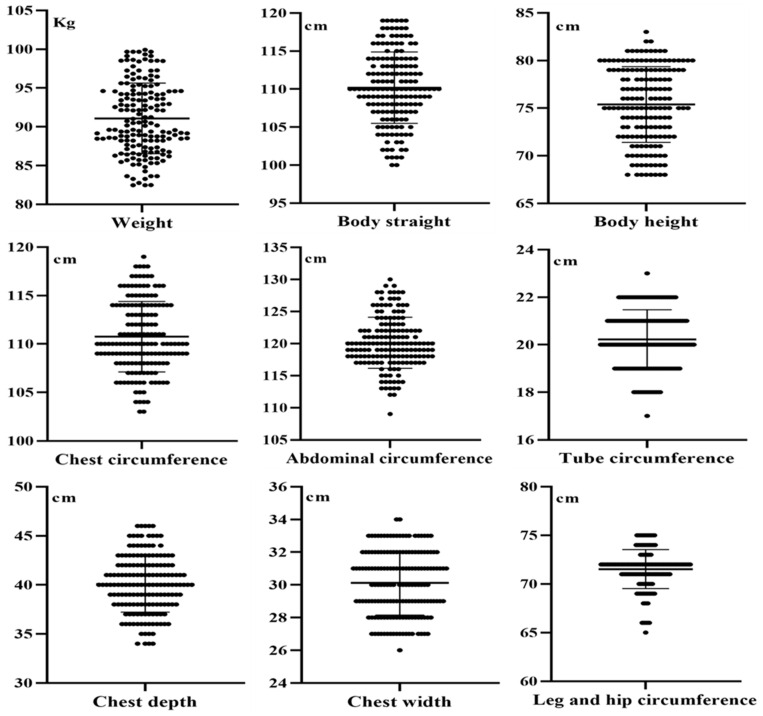
Distribution area of growth data for 165 Xiangsu pigs.

The mean, maximum, minimum, and standard deviation values for nine growth traits (withers height, body weight, body length, chest width, chest depth, chest girth, abdominal girth, rib girth, and hind limb girth) in 165 fattening Xiangsu pigs were calculated. The results are presented in Table 2. Analysis of the distribution of these growth traits (Figure 5) revealed variability among individual data points, with a significant clustering around the mean, in line with the principles of randomness and variability.

### 3.5. Analysis of Population Genetic Diversity

Table 3 presents the genotype frequencies and genetic parameters for five mutation sites in the ENPP1 gene of pigs. Genetic analysis was conducted on these five SNPs, and the results of the Chi-square (χ^2^) test are shown in the table. The χ^2^ values for the SNPs g.64275 T→C, g.64429 G→A, g.64527 T→C, g.64850 T→C, and g.64911 G→A were 0.953, 3.802, 0.014, 0.213, and 0.106, respectively, indicating that the genotype distributions were in Hardy–Weinberg equilibrium (*p*-value > 0.05). As shown in Table 4, the effective allele numbers for these five SNPs ranged from 1.581 to 1.994, with heterozygosity values of between 0.367 and 0.499 and homozygosity values of between 0.502 and 0.633. In all cases, homozygosity was higher than heterozygosity. The polymorphic information content (PIC) values for these SNPs were 0.300, 0.357, 0.374, 0.368, and 0.360 (all falling within the range of 0.25 < PIC < 0.5), indicating moderate polymorphism.

### 3.6. Correlation Analysis Between Genotype and Growth Traits

The relationship between five mutation sites in the ENPP1 gene and nine growth traits was examined in 165 Xiangsu hybrid pigs. The association analysis of different genotypes at the ENPP1 mutation sites (g.64275T→C, g.64429G→A, g.64527T→C, g.64850T→C, and g.64911G→A) with growth traits is presented in Table 5. For the g.64275T→C site, pigs with the CC genotype showed significantly better performance in weight, body height, and chest depth compared to those with the TC genotype (*p*-value < 0.05), and a significantly better chest circumference (*p*-value < 0.01). At the g.64429G→A site, the AA genotype exhibited a highly significant improvement in chest depth over the GG genotype (*p*-value < 0.01). For the g.64527T→C site, the CC genotype had significantly better chest depth than the TT genotype (*p*-value < 0.05).

## 4. Discussion

China is home to the most diverse range of indigenous pig breeds, which are regionally categorized into types such as Central China, North China, Jiang-Hai, Southwest, South China, and Plateau. The Congjiang Xiang pig, a small-sized breed from Guizhou, belongs to the Southwest type. It is well regarded for its tender meat and robust stress resistance; however, its slow growth significantly impedes the local pig farming industry’s economic development [20,21,22]. The Xiangsu hybrid pig inherits the Congjiang Xiang pig’s desirable meat qualities while demonstrating substantial improvements in body size and reproductive performance. Consequently, the development of the Xiangsu pig breed plays a pivotal role in advancing the pig farming industry in Guizhou. Studies have shown that the ENPP1 gene is involved in various physiological processes across multiple tissues. The products of the ENPP1 protein catalyze extracellular signaling molecules that regulate physiological functions or serve as ligands for receptors on the cell surface and within cells [23,24,25]. ENPP1 also regulates bone and soft tissue mineralization by generating pyrophosphate and is involved in bone remodeling [15,26]. ENPP1 deficiency is associated with ectopic calcification in multiple tissues, neointimal proliferation, growth and developmental disorders, and skeletal deformities [27,28,29]. Since bone and muscle functions are closely interconnected, they interact through osteogenic and myogenic factors. Osteoporosis is often accompanied by a loss of muscle mass or is linked to postpartum paralysis in sows [30]. Recent studies have demonstrated that ENPP1 is expressed in various tissues of Xiangsu pigs at different stages, with significant variation in expression levels, which aligns with previous findings [31,32]. Therefore, investigating the association between ENPP1 gene mutations and growth performance in pigs is of considerable value. To date, however, no studies have addressed this topic. This study aims to explore the correlation between ENPP1 gene SNPs and growth performance in Xiangsu pigs, which holds important research implications.

In mammalian genetics, nucleotide sequence alterations—resulting from artificial interventions, natural selection, or genetic drift—can influence an organism’s genetic traits. Generally, higher polymorphic information content (PIC) corresponds to greater genetic diversity within a population [33,34]. This study analyzed the SNPs in the ENPP1 gene in the Xiangsu pig population. Five novel SNP sites were identified in the ENPP1 gene, all exhibiting moderate polymorphism (0.25 < PIC < 0.50), with no deviations from Hardy–Weinberg equilibrium. These findings suggest that mutations at these sites may impact the genetic equilibrium of the population and potentially influence evolutionary processes, indicating their selective value for breed improvement [33,35]. Sequence alignment revealed that g.64527T→C is located in the exon region, while the remaining four sites are within intronic regions. Previous research has shown that intronic variations can disrupt mRNA expression, protein function, and splicing in eukaryotes [36,37,38]. Thus, intronic mutations may also affect growth traits and serve as valuable genetic markers. Although no amino acid changes were observed at the identified sites, SNPs in the intronic and 3′-UTR regions can still regulate gene expression [39,40,41]. These regulatory and functional sites strengthen the potential role of the ENPP1 gene in modulating pig growth performance. To further investigate the impact of each SNP on growth traits, we performed genotype and haplotype association analyses. The results indicated that g.64275T→C, g.64429G→A, and g.64527T→C significantly influenced growth performance in Xiangsu pigs. Specifically, the g.64275T→C site was associated with withers height, chest circumference, and chest depth (*p* < 0.05, *p* < 0.01), while g.64429G→A and g.64527T→C were both linked to chest depth (*p* < 0.05, *p* < 0.01), highlighting their potential as candidate markers for this trait. Additionally, structural and functional predictions of the ENPP1 protein revealed that pigs are most distantly related to poultry in evolutionary terms. Fifteen significant amino acid sequences were identified across eight species, suggesting functional similarities at the supersecondary structure level. Moreover, ENPP1 exhibits considerable conservation across species. Previous studies have also suggested that polymorphisms in the 3′ UTR binding region of the ENPP1 gene are associated with increased ENPP1 protein levels in skeletal muscles [42].

The aforementioned studies demonstrate a significant correlation between the ENPP1 gene and production performance in livestock, highlighting its potential as a key reference gene for breeding programs. Our results also suggest that polymorphisms in the ENPP1 gene may be closely linked to growth performance in Xiangsu pigs, justifying further investigation in future breeding studies.

## 5. Conclusions

In summary, this study identified five novel SNPs in the ENPP1 gene of pigs and analyzed their association with growth performance. The results indicate that the SNPs g.64275T→C, g.64429G→A, and g.64527T→C significantly influence growth traits. These findings offer valuable insights for molecular marker-assisted selection and the enhancement of growth performance in Xiangsu pigs.

## Figures and Tables

**Table 1:** Primers for the porcine ENPP1 gene.

Genes	Product Size (bp)	Primer Sequence (5′-3′)	Tm (°C)
ENPP1-Exon1	808	F: AGTGCCCGAAATCAGACAGGAAG R: CCTTGTCGGCTTTTCCTAAGACC	56
ENPP1-Exon2	241	F: GCACACATTGTATGACCAGCACTA R: CGAATCTCAGTCACATTCTGAAGG	57
ENPP1-Exon3	992	F: CAGCAATGCCAGATCCTTAACCC R: CCAGTCACTGTTGGAGAACTCAGAA	57
ENPP1-Exon4	875	F: ACTCGGACTTAGGAAATAGCCCA R: TACCTGTGCTAAACCCAGCTAAGAC	63
ENPP1-Exon5	644	F: GTGGATAGAAGGAGGGTTGTCAGTT R: GAGTTCTCTTCTTGGCTCAGCAGTT	65
ENPP1-Exon6	712	F: CTTTAGACGACATGCCGACATCAG R: GGCTACAGCTCTGATTAGACCCCTA	62
ENPP1-Exon7	645	F: CTTTTCTTAGCACTGGTGCCAAAG R: GAACACTGTAAACCAGCCGAAATG	62
ENPP1-Exon8	665	F: ACCAAAGCAAAGAGGAGTTTGCTC R: ACCCAGCTCTGGAATAAACTCCAC	63
ENPP1-Exon9	325	F: CTTAAGAGGAAAGCACTGGTGTTG R: ACAGGTCTCATCGTTCAGACAACA	61
ENPP1-Exon10	486	F: GCGTCCACTTCCCAGTTTTACTA R: TCAGAGCCAAGAGCAAAGTCTTC	57
ENPP1-Exon11	408	F: AGGAATGATACCCCAGTGTTGTG R: CCACTGGTTCAACTTAGCACTGTAG	59
ENPP1-Exon12	475	F: ATGTGCCATTCTGTGCCCTTGT R: CGGAGCCACAACAGGAACTCTTAT	59
ENPP1-Exon13	896	F: CTCTGGATATATGTCCAGGAATGGG R: TCATCTCTTCCAGCATTCCTCCTT	57
ENPP1-Exon14	598	F: GGCTGAGTGGTTCCGAAAAATCT R: AACCAGAGCCACAGTGGTGAGAAT	59
ENPP1-Exon15	543	F: AACTAGGGACTGAATCTAGGCATCC R: GCCAAGGTTAAGAGACCCTGAGATA	59
ENPP1-Exon16	843	F: TAGCATGGTCAGGGTTCAAAGTC R: TCTCACTCTGCCCAACATACACA	60
ENPP1-Exon17	363	F:CATGGTAAATGTACACCAGGGGTT R:CCTGACCTATGACCTCCTTTTGAT	62
ENPP1-Exon18	702	F: CAAGGTCTGTCTGTATTCAGCTACC R: AGGTCTGCCTGTGTGACTAGAATTG	56
ENPP1-Exon19	792	F: GGGAGAGATCTAGCTTAGTCCTGGA R: TCTGTTTCATCAGGTGGTCCTCC	56
ENPP1-Exon20	977	F: CGAATCCGACTAGGAACCATGAG R: CGGGGTTTCAAAGTCACGTAGATAG	57
ENPP1-Exon21	791	F: GCATATGGAGGTCACCAGCTTATG R: TAGCTCCAATTAGACCCCTAGCCT	59
ENPP1-Exon22	917	F: TAGTTGAAATGAGGCAGAGGAGG R: GTTATCCGGGCTGATGGTCAATA	57
ENPP1-Exon23	677	F: GGGATGCTTGAAGTACAGCCGAT R: GACAAGATCGTAACAGCAGCCACA	57
ENPP1-Exon24	557	F: CAGTCTGGCAGGAAATATTGAGG R: GCCATACGTAGCCAGTGTAAGGTTT	57
ENPP1-Exon25	617	F: TGATGAGAACGGAGGAGATGGA R: AAGGGTTTGCCAGCATCAGAGA	61
ENPP1-Exon26	1006	F: GCAGAAGTGAATGTTGTCTGGTCAC R: CATCAGAATGGATTAGGCAGAGGA	59
ENPP1-Exon27	1478	F: CCATACCCTTTAGTACCAACCTGTG R: GATGCTCTACATAGAGGCAACTGTG	61
ENPP1-qPCR	109	F: GAGAACATTTGGGAACTGTCGC R: GCTGCAAGTCCATATCCGTTCT	63
GAPDH-qPCR	169	F: TTGTGATGGGCGTGAACC R: GTCTTCTGGGTGGCAGTGAT	58

**Table 2:** Growth performance data of Xiangsu pigs.

Index	W/kg	B S/cm	B H/cm	C C/cm	A C/cm	T C/cm	C D/cm	C W/cm	L H C/cm
Mean	91.08	110.18	75.39	110.75	120.12	20.22	40.09	30.12	71.53
Max	99.92	119	83	119	130	23	46	34	75
Min	82.47	100	68	103	109	17	34	26	65
SD	4.55	4.70	3.97	3.65	3.99	1.25	2.88	1.96	2.01

Note: W: weight/kg, B S: body straight/cm, B H: body height/cm, C C: chest circumference/cm, A C: abdominal circumference/cm, T C: tube circumference/cm, C D: chest depth/cm, C W: chest width/cm, L H C: leg and hip circumference/cm.

**Table 3:** The genotype frequency and gene frequency of ENPP1 in Xiangsu pigs.

SNPs	Genotypic Frequencies			Allelic Frequency		χ^2^
g.64275T→C	TT	TC	g.64275T→C	T	C	0.953
	0.073(12)	0.339(56)	0.588(97)	0.242	0.758	
g.64429G→A	GG	GA	g.64429G→A	G	A	3.802
	0.436(72)	0.394(65)	0.170(28)	0.633	0.367	
g.64527T→C	TT	TC	g.64527T→C	T	C	0.014
	0.424(70)	0.4(66)	0.176(29)	0.624	0.376	
g.64850T→C	TT	TC	g.64850T→C	T	C	0.213
	0.333(55)	0.503(83)	0.164(27)	0.585	0.415	
g.64911G→A	GG	GA	g.64911G→A	G	A	0.103
	0.358(59)	0.491(81)	0.152(25)	0.603	0.397	

Note: χ^2^_0.05_ < 5.99; χ^2^_0.01_ < 9.21, sample size in parentheses. Note: HE, heterogeneity; HO, pureness; NE, effective allele; PIC, polymorphic information content; PIC < 0.25 implies low polymorphism; 0.5 > PIC > 0.25 denotes moderate polymorphism; PIC > 0.5 suggests high polymorphism; χ^2^ < 5.99 denotes Hardy–Weinberg equilibrium; χ^2^ > 5.99 signifies Hardy–Weinberg disequilibrium.

**Table 4:** Genetic diversity of SNP loci in the ENPP1 gene of Xiangsu pigs.

SNPs	He	Ho	Ne	PIC
g.64275T→C	0.367	0.633	1.581	0.300
g.64429G→A	0.464	0.536	1.867	0.357
g.64527T→C	0.499	0.502	1.994	0.374
g.64850T→C	0.456	0.514	1.994	0.368
g.64911G→A	0.479	0.521	1.919	0.364

Note: Ho represents homozygosity, He represents heterozygosity, Ne represents effective allele number, and PIC represents peptide information content. PIC < 0.25 indicates low polymorphism, 0.25 < PIC < 0.50 indicates moderate polymorphism, and PIC > 0.5 indicates high polymorphism.

**Table 5:** Association analysis of three SNPs in ENPP1 and the growth trait.

SNPs	Genotype	W/kg	B S/cm	B H/cm	C C/cm	A C/cm	T C/cm	C D/cm	C W/cm	L H C/cm
g.64275T→C	TT(12)	89.91 ± 4.41	111.25 ± 4.99	74.67 ± 3.94	109.33 ± 1.92	118.58 ± 2.15	20.42 ± 0.67	38.92 ± 2.47	29.92 ± 2.31	71.58 ± 2.07
	TC(56)	90.13 ± 4.51 ^a^	109.65 ± 4.80	74.58 ± 3.95 ^a^	109.85 ± 3.31 ^A^	119.49 ± 3.82	20.00 ± 1.17	39.45 ± 2.87 ^a^	30.07 ± 1.94	71.38 ± 1.88
	CC(97)	91.76 ± 4.50 ^b^	110.35 ± 4.62	75.93 ± 3.94 ^b^	111.43 ± 3.85 ^B^	120.65 ± 4.17	20.33 ± 1.33	40.60 ± 2.85 ^b^	30.17 ± 1.95	71.61 ± 2.09
g.64429G→A	GG(72)	90.38 ± 4.54	109.94 ± 5.05	74.68 ± 3.89	110.28 ± 3.60	119.78 ± 4.14	20.17 ± 1.20	39.50 ± 2.88 ^A^	30.01 ± 2.00	71.65 ± 1.82
	GA(64)	91.73 ± 4.72	110.14 ± 4.48	75.89 ± 4.19	110.89 ± 3.61	120.18 ± 3.75	20.28 ± 1.32	40.26 ± 2.87	30.12 ± 1.91	71.48 ± 2.34
	AA(29)	91.39 ± 4.02	110.89 ± 4.37	76.04 ± 3.46	111.64 ± 3.79	120.82 ± 4.16	20.25 ± 1.24	41.25 ± 2.58 ^B^	30.39 ± 2.04	71.36 ± 1.68
g.64527T→C	TT(70)	90.50 ± 4.51	109.97 ± 5.06	74.94 ± 3.86	110.36 ± 3.60	119.87 ± 4.16	20.17 ± 1.20	39.54 ± 2.91 ^a^	29.99 ± 2.00	71.74 ± 1.78
	TC(66)	91.50 ± 4.80	110.06 ± 4.50	75.67 ± 4.25	110.79 ± 3.65	120.11 ± 3.77	20.27 ± 1.32	40.24 ± 2.85	30.20 ± 1.88	71.38 ± 2.36
	CC(29)	91.53 ± 4.02	110.97 ± 4.31	75.83 ± 3.58	111.62 ± 3.73	120.72 ± 4.12	20.24 ± 1.21	41.10 ± 2.65 ^b^	30.28 ± 2.10	71.38 ± 1.66
g.64850T→C	TT(55)	90.81 ± 4.39	111.04 ± 4.53	75.36 ± 3.70	110.67 ± 3.39	120.04 ± 3.57	20.18 ± 1.11	40.31 ± 2.77	30.20 ± 2.12	71.27 ± 1.84
	TC(83)	91.18 ± 4.55	109.63 ± 4.50	75.34 ± 4.23	110.45 ± 3.71	119.82 ± 4.17	20.19 ± 1.30	39.76 ± 2.95	30.08 ± 1.88	71.61 ± 2.24
	CC(27)	91.33 ± 4.98	110.15 ± 5.53	75.59 ± 3.84	111.85 ± 3.88	121.19 ± 4.18	20.41 ± 1.37	40.70 ± 2.84	30.07 ± 1.96	71.81 ± 1.57
g.64911G→A	GG(59)	90.98 ± 4.40	110.85 ± 4.56	75.27 ± 3.73	110.63 ± 3.41	119.93 ± 3.54	20.20 ± 1.11	40.36 ± 2.70	30.27 ± 2.07	71.31 ± 1.79
	GA(81)	91.11 ± 4.51	109.65 ± 4.46	75.44 ± 4.21	110.53 ± 3.69	119.89 ± 4.17	20.21 ± 1.31	39.75 ± 2.99	30.04 ± 1.87	71.62 ± 2.27
	AA(25)	91.22 ± 5.15	110.32 ± 5.69	75.48 ± 3.87	111.76 ± 4.02	121.28 ± 4.34	20.32 ± 1.38	40.60 ± 2.92	30.04 ± 2.03	71.80 ± 1.61

Note: a, b indicate significant differences between different genotypes (*p*-value < 0.05); A, B indicate extremely significant differences between different genotypes (*p*-value < 0.01).
